# Toll-like Receptor 8 (TLR8) Is Expressed in Mouse Hippocampal Neurons and Modulates Neuronal Excitability

**DOI:** 10.3390/ijms27146232

**Published:** 2026-07-13

**Authors:** Julian Fenkart, Alice Santagostino, Stephanie Seidlberger, Armin Schmuck, Katharina Donat, Meinrad Drexel, Sandra Santos-Sierra

**Affiliations:** Institute of Pharmacology, Medical University of Innsbruck, Peter Mayr Str. 1, 6020 Innsbruck, Austria; julian.fenkart@i-med.ac.at (J.F.);

**Keywords:** TLR, neuron, excitability, antagonist

## Abstract

Toll-like receptors (TLRs) are essential in the innate immune response. Furthermore, neuronal TLRs have been involved in regulating neuronal dendritic outgrowth and excitability. However, a deeper understanding of neuronal-TLR function is essential. We investigated the expression and role of TLR8 using mouse postnatal hippocampal neuronal cultures. We assessed TLR8 (and TLR7) expression by immunocytochemistry and TLR8 functionality via electrophysiological recordings in a high-density multi-electrode array system and calcium flux imaging. Pathway activation following fast TLR8 stimulation was performed via Western blot. TLR8 and TLR7 are expressed in postnatal hippocampal neurons at DIV14 and in neurosphere-derived neurons. TLR8 expression was detected in the cell body and dendrites of GABAergic, parvalbumin-expressing interneurons. Stimulation with the TLR8 agonist TL8-506 led to increased neuronal activity in the short term and enhanced neuronal synchronization in short- and long-term recordings. The calcium flux induced by TLR8 was reduced by blocking NMDA receptors with D-AP5. TLR8 stimulation did not lead to IkB-α degradation. Our findings demonstrate that TLR8 and TLR7 are expressed in mouse postnatal hippocampal neurons. TLR8 activation leads to increased neuronal excitability that was decreased by NMDA antagonism. However, acute TLR8 stimulation failed to robustly activate the canonical TLR signaling pathway. We propose that TLR8 activation might lead to the enhancement of excitatory NMDA receptors.

## 1. Introduction

Toll-like receptors (TLRs) were initially discovered in the fruit fly *Drosophila melanogaster*, where they play pivotal roles in establishing the dorsoventral axis providing immune defense against fungal infections [1]. Subsequently, TLR orthologues were identified in mammals, emerging as key components of the innate immune system [2]. As pattern-recognition receptors (PRRs), TLRs expressed in immune cells, such as macrophages and dendritic cells, can recognize pathogen-associated molecular patterns (PAMPs) derived from pathogens and danger-associated molecular patterns (DAMPs) from stressed or dying host cells. This recognition initiates downstream signaling pathways that lead to the production of pro-inflammatory cytokines (e.g., TNF-α, IFN-α) [3]. TLRs can be categorized into two major classes based on their cellular localization: TLR1, TLR2, TLR4, TLR5, and TLR6 are expressed on the plasma membrane, while TLR3, TLR7, TLR8, and TLR9 are found in endosomes and require ligand internalization to initiate an inflammatory response [4]. Specifically, TLR8 has been shown to recognize viral single-stranded RNA (ssRNA) and endogenous microRNAs within exosomes [5,6].

Although the role of TLRs in immune cells is well-established, their functions in the central nervous system, particularly in neurons, remain largely unexplored. Previous studies have indicated that TLR signaling in neurons regulates non-immune functions like neuronal morphology and proliferation [7,8]. For instance, TLR8 has been identified as a negative regulator of dendritic outgrowth in cortical neurons [9]. Additionally, TLR4 signaling has been implicated in modulating neuronal excitability in epilepsy [10]. In this context, a danger signal released from hyperexcitable neurons activates the TLR4 signaling pathway, leading to enhanced Ca^2+^ influx through the NMDA receptor, ultimately resulting in heightened neuronal excitability [11]. Further studies have shown that blocking the TLR4 signaling axis can attenuate neuronal hyperactivity in a kainate-induced epilepsy model [12]. Moreover, it has been shown that stimulation of TLR3 with poly(I:C) and co-stimulation with IFN-α (mouse model of depression) leads to increased neuronal AMPAR1 levels in vitro, while ex vivo treatment results in stable levels of AMPAR1 (although less phosphorylated). Simultaneously, the scaffolding protein PSD95 levels are decreased after the in vitro treatment. This leads to a structural uncoupling between AMPAR1 and PSD95 resulting in impaired neuronal excitability and weaker synaptic connections [13].

Building on the insights from previous research, we postulated that other TLRs, apart from TLR4, might also influence neuronal excitability upon activation. Accordingly, we reported the expression of TLR8 in various types of neurons in mouse hippocampal brain slices [14]. Given that the hippocampus is one of the most epileptogenic regions in the brain, in the current study, we delved into the functional role of neuronal TLR8 and investigated whether its activation in postnatal hippocampal neuronal cultures might modulate neuronal excitability. By exploring the impact of TLR8 activation on neuronal function, we aim to enhance our understanding of the intricate interactions between the immune and nervous systems and identify potential avenues for novel therapeutic interventions in neurological disorders characterized by aberrant neuronal activity.

## 2. Results

### 2.1. TLR8 Expression in Mouse Postnatal Hippocampal Neurons

**Figure 1:**
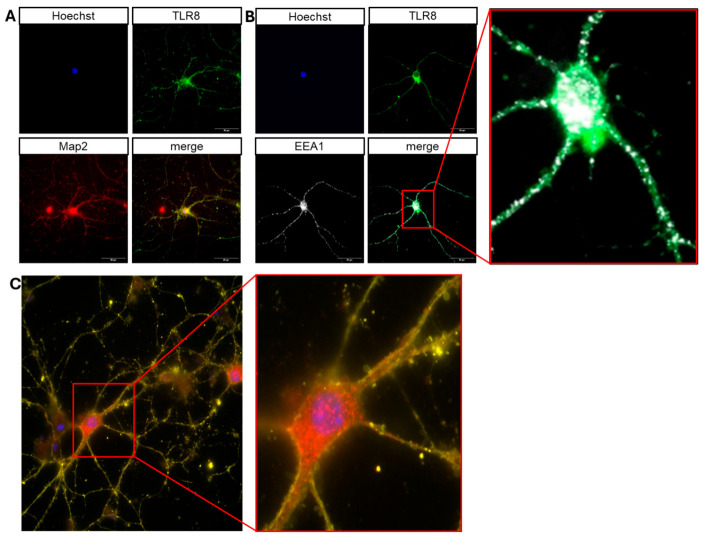
TLR8 expression and localization in mouse postnatal hippocampal neurons. (**A**) Immunocytochemistry of TLR8 in cultured postnatal hippocampal neurons (DIV14) and the neuronal marker Map2. (**B**) Immunocytochemistry of TLR8 and the endosomal marker EEA1 in cultured postnatal hippocampal neurons (DIV14). (**C**) Immunocytochemistry of TLR8 (here red for a better contrast) together with the cytoplasmic membrane marker CellBrite (yellow). Scale bar 50 μm. Cell nuclei were stained with HOECHST.

Previous studies have indicated that, during postnatal development, TLR8 expression in neurons is predominantly confined to the soma [9]. To verify these findings, we conducted immunostainings of TLR8 and co-staining with the neuronal marker Map2 in postnatal hippocampal neurons at DIV14 (days in vitro 14) (Figure 1A). Our results showed that TLR8 exhibited its highest expression level in the soma of the neurons. However, contrary to the observations of Ma et al. [9], TLR8 expression in postnatal neurons was not exclusively restricted to the soma but was distributed throughout the entire cell (see Figure 1A). Due to this discrepancy, we sought to validate the specificity of the TLR8 antibody used in our experiments. Hence, we performed immunolabeling with three additional TLR8 antibodies, which yielded the same expression pattern as previously observed (Figure S1A). Additionally, to get preliminary information about the localization of TLR8, we performed immunocytochemistry with the early endosomal marker EEA1 (Figure 1B) and with the cytoplasmic membrane dye CellBrite (Figure 1C). TLR8 exhibited the punctate, focal-expression pattern typical of endosomal localization, while concurrently displaying diffuse, extra-foci expression along the soma and dendritic processes (Figure 1B).

**Figure 2:**
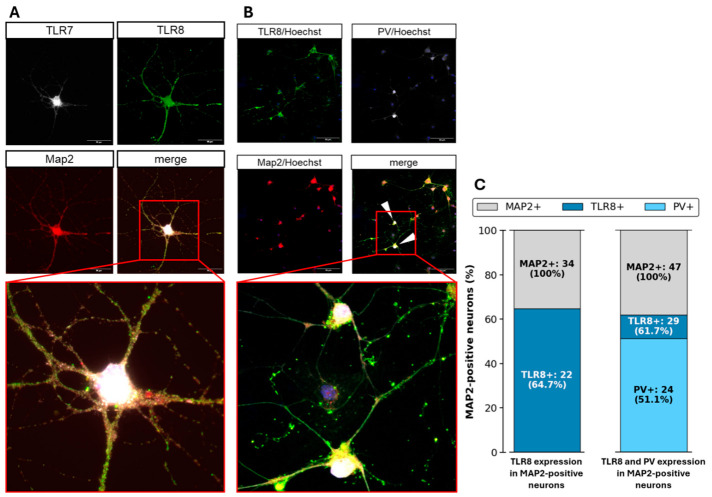
TLR8 and TLR7 neuronal expression. (**A**) Immunocytochemistry of TLR8 (green) and TLR7 (gray) together with the neuronal marker Map2 (red) in cultured postnatal hippocampal neurons (DIV14). (**B**) Immunocytochemistry of TLR8 (green) in cultured postnatal hippocampal neurons at DIV14, along with Parvalbumin (gray) and Map2 (red). Nuclei were visualized with HOECHST staining (blue). Scale bar 50 μm. (**C**) Quantification of TLR8 positive and parvalbumin positive neurons. Numbers within the bars indicate the absolute number of positive stained cells and the corresponding percentage relative to the total number of Map2-positive neurons analyzed.

Both TLR7 and TLR8 act as receptors for single-stranded RNAs (ssRNAs) and exhibit some homology in their amino acid sequences [15,16,17]. Given this similarity, we proceeded to compare the expression patterns of TLR7 and TLR8 in postnatal hippocampal neurons using triple-immunocytochemistry of TLR7, TLR8, and Map2 (Figure 2A). Validation of antibody specificity is provided in Figure S1B,C.

The results demonstrated that both TLR8 and TLR7 were expressed in postnatal hippocampal neurons at DIV14 (Figure 2A and Figure S2A,B). Moreover, the expression patterns of TLR7 and TLR8 showed a similar intracellular distribution, although they did not co-express in all the regions. These findings contrast with the observations made by Ma et al. [9], who did not detect TLR7 expression in neurons. Furthermore, the expression of both TLR7 and TLR8 was detected in neurons derived from neurospheres matured for two weeks (Figure S3). In contrast to the established expression of TLR3 in neural stem/progenitor cells [18], the presence or TLR8 and TLR7 in these populations has not yet been investigated.

In previous findings, our group observed a strong expression of TLR8 in a specific type of inhibitory interneurons in hippocampal mouse brain slices, specifically the parvalbumin (PV) interneurons, while other neurons such as granule cells of the dentate gyrus or hippocampal pyramidal cells showed only weak TLR8 expression [14]. To confirm the co-expression of TLR8 with PV on hippocampal neuronal cultures, we performed immunocytochemistry experiments in which we stained TLR8, PV, and the neuronal marker Map2. As shown in Figure 2B,C and Figure S4, TLR8 expression was indeed found in PV-expressing neurons (51.1%), although not exclusively, confirming our previous findings in mouse brain sections. In astrocytes we did not observe TLR8 expression (Figure S5D).

### 2.2. TLR8 Contributes to Neuronal Excitability In Vitro

After confirming the expression of TLR8 in hippocampal postnatal neuronal cultures, we aimed to investigate its impact on neuronal excitability. To achieve this, primary hippocampal cultures at DIV14 were exposed to the agonist TL8-506 a derivative of VTX-2337 [19]. The cultures were grown on a high-density multi-electrode array (HD-MEA) chip, enabling real-time monitoring of their electrophysiological response. We first validated the experimental setup utilizing the neuronal cultures grown on MEA chips by using the GABA_A_-receptor antagonist picrotoxin and various anti-seizure drugs (Figure S6).

**Figure 3:**
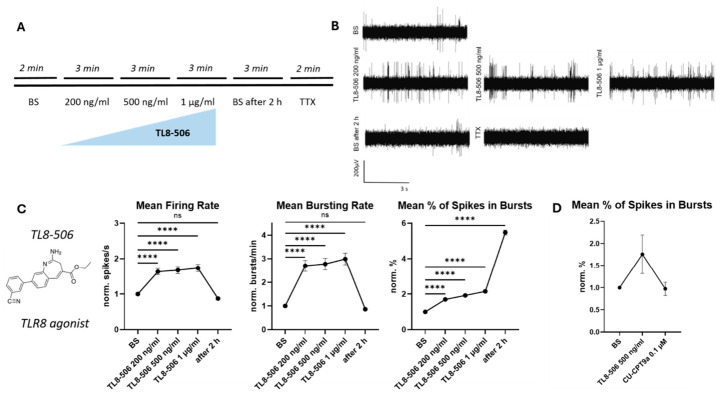
Stimulation with the selective TLR8 agonist TL8-506 increases activity of mouse postnatal hippocampal cultures. (**A**) Scheme of the MEA recording protocol for TLR8 stimulation in hippocampal neuronal cultures. Baseline activity (BS) was recorded for 2 min, followed by cumulative stimulation with TL8-506 at concentrations. Neuronal cultures were then incubated with TL8-506 (1.0 µg/mL) at 37 °C with 5% CO_2_ for 2 h. Baseline activity was again measured for 3 min after incubation with the agonist. To exclude electrodes that gave a false positive signal, the voltage-gated sodium channel blocker tetrodotoxin (TTX) (1 µM) was applied at the end of the recordings. (**B**) Representative traces of one HD-MEA recording, showing a single channel. (**C**) Chemical structure of the TLR8 selective agonist TL8-506 (Invivogen, San Diego, CA, USA) and analyzed parameters of MEA recording after TLR8 stimulation. Increased neuronal activity is reflected by augmented firing rate (spikes/s), bursting rate (bursts/min), and higher network synchrony (mean % of spikes in bursts) compared to unstimulated baseline activity of the neurons. Results are displayed as mean ± SEM, with the number of independent postnatal neuronal cultures (*N*) being 5, and the number of recorded channels for each recording being 452, 805, 1461, 1150, and 1093. The data were analyzed using one-way ANOVA, and normalization was performed by dividing the raw values through the mean baseline activity of the MEA chip. (**D**) Inhibition of TLR8 with CU-CPT9a decreased neuronal activity back to baseline levels. Results are displayed as mean ± SEM, with the number of independent postnatal neuronal cultures (*N*) being 2 and the number of recorded channels being 2864 and 2161. **** *p* ≤ 0.0001; ns, non-significant, *p* ≥ 0.05.

MEA recordings were initiated by measuring the baseline activity of the neurons for two minutes (Figure 3A). Subsequently, TLR8 was stimulated with TL8-506 at increasing concentrations (0.2–1.0 µg/mL). The effect of TLR8 stimulation on neuronal activity was recorded for three minutes under each concentration of TL8-506. To evaluate the long-term effects of TLR8 stimulation, neuronal cultures were incubated for two hours after the last application of TL8-506 (1.0 µg/mL), and baseline activity was measured again for 3 min followed by tetrodotoxin (TTX) incubation to exclude electrodes that gave a false positive signal due to electrical noise.

The MEA recordings demonstrated that stimulation of TLR8 with TL8-506 significantly increased the activity of hippocampal neurons (Figure 3B,C). Immediately after applying the lowest concentration of TL8-506 (200 ng/mL), the neuronal cultures displayed an enhanced activity within a few seconds, as evident from the higher mean firing rate (increased by 70%) and mean bursting rate (increased by 190%) compared to the baseline activity of the unstimulated neurons. Moreover, neuronal activity increased with escalating concentrations of the TLR8 agonist, reaching its peak with the concentration 1.0 µg/mL of TL8-506 (Figure 3C).

Interestingly, the mean percentage of spikes in bursts increased after the administration of TL8-506, both in the short and long term (Figure 3C). This parameter measures how many spikes are organized in bursts rather than being fired as single spikes, indicating that the network’s activity became more temporally clustered and synchronous.

Further, to validate that the observed increase in neuronal activity after TL8-506 application was indeed TLR8 activity-dependent, we tested the effect of a selective TLR8 antagonist, CU-CPT9a, in the functional MEA recording (Figure 3D). The results demonstrated that treatment with CU-CPT9a reversed the increased activity induced by TL8-506 back to baseline levels.

Notably, the neuronal cultures used in our study, following the protocol described by Moutin et al. [20], were heterogeneous, comprising approximately 45% neurons, 53% astrocytes, 1% microglia, and 1% oligodendrocytes (Figure S5). As a result, we questioned whether the observed increase in excitability after TLR8 stimulation might be driven by the activation of inflammatory pathways in co-cultured glial cells or by an increase in activity of ion channels in neurons. The short-term stimulation with TL8-506 (nine minutes) led to augmented excitability of the neurons, as evidenced by increased mean firing rate and bursting rate compared to baseline levels. In contrast, during the two-hour long-term stimulation, there was no significant increase in neuronal activity compared to the baseline levels. Both, the mean firing rate and bursting rate returned to their original baseline levels after the extended application of TL8-506 (1 µg/mL; Figure 3C). However, neuronal synchronization (percentage of spikes in bursts) remained increased. These findings suggest that the initial increase in neuronal activity observed after TLR8 activation might be caused by changes in ion channel activity [21]. These findings reveal that the stimulation of TLR8 with TL8-506 in postnatal neuronal cultures results in increased neuronal activity in the short-term and increased neuronal synchronization both in the short and long term. These changes in excitability exhibit similarities with the characteristics observed in rat primary neuronal cultures and in brain slices treated with pro-convulsive drugs [22].

**Figure 4:**
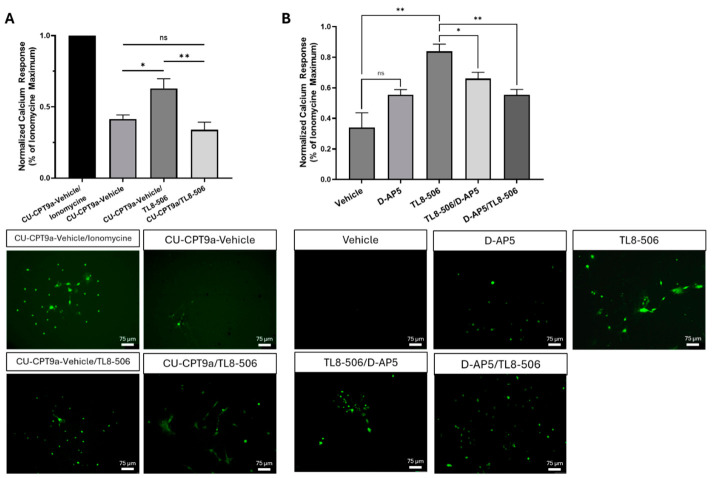
Calcium imaging of primary hippocampal neurons following TL8-506 stimulation and TLR8 inhibition. (**A**) The activity of TL8-506 was inhibited by CU-CPT9a or the cells were treated with the specific vehicle used to dissolve CU-CPT9a. For comparison the activity obtained with ionomycin is indicated. *N* = 3. (**B**) Effect of D-AP5 on TL8-506-induced Ca^2+^-responses. The data was normalized within each plate to the respective ionomycin values. *N* = 3. Bars indicate mean ± SEM. Statistical analyses were performed using repeated measures one-way ANOVA with planned pairwise comparisons as indicated. ns, non-significant *p* ≥ 0.05; * *p* < 0.05; ** *p* < 0.01.

Previous studies have demonstrated that TLR4 activation increases neuronal excitability by enhancing NMDA-mediated Ca^2+^ influx [11]. Also, TLR4 signaling enhances AMPAR currents leading to post-traumatic epileptogenesis in rats subjected to brain injury [23]. To explore whether the observed neuronal excitability following TLR8 activation might be explained through a similar mechanism, we stimulated neuronal cultures with TL8-506 and incubated them with the fluorophore Fluo-4 (Figure 4A). Additionally, we treated the cells with the TLR8 antagonist CU-CPT9a or its specific vehicle, and we used the ionophore ionomycin as a positive control of Ca^2+^ mobilization. TLR8 stimulation induced a marked increase in fluorescence as it was also observed with ionomycin. In contrast, CU-CPT9a reduced significantly the observed fluorescence to basal levels. The observe effect might be at least TLR8-mediated since CU-CPT9a is described as a selective TLR8 antagonist.

**Figure 5:**
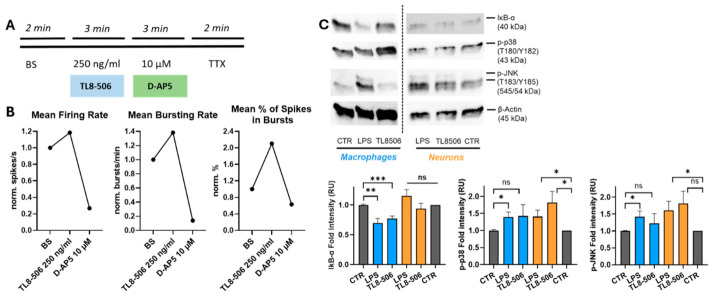
Inhibition of NMDA receptor activity and downstream pathways induced by TLR8 stimulation in hippocampal neurons. (**A**) Scheme of MEA recording to investigate the effect of TLR8 stimulation and inhibition of NMDA receptors in hippocampal neuronal cultures. Baseline activity (BS) was recorded for 2 min, followed by stimulation of the neuronal culture with 250 ng/mL of the TLR8 agonist TL8-506 for 3 min. Subsequently, the culture was treated with the NMDA receptor antagonist D-AP5 at 10 µM for 3 min. The voltage-gated sodium channel blocker tetrodotoxin (TTX) (1 µM) was applied at the end of the recordings to exclude false positive electrode signals. (**B**) Analysis of MEA recording data in postnatal neuronal cultures (DIV14, cell density 7.5 × 10^4^ cells/MEA BioChip). Results are displayed as mean ± SEM, with data obtained from one postnatal neuronal culture. The number of recorded channels was *n* = 882. (**C**) Hippocampal neuronal cultures at DIV14 (cell density of 1.6 × 10^5^ cells/well) were stimulated with TL8-506 and the degradation of IkB-α, and phosphorylation of the MAPKs p38 and JNK was assessed in comparison to mouse macrophages (cell density of 1.5 × 10^5^ cells/well). LPS (lipopolysaccharide 5 µg/mL; TLR4) was used as a control of stimulation. The broken line separates samples run in the same gel; however, the intensity of the neuronal samples was increased for comparison (linear change; gamma = 1). The data were normalized to the β-Actin levels and to the unstimulated control sample (CTR). Student’s *t*-test was used to compare the stimulation obtained with TL8-506 versus the unstimulated sample. ns, non significant, *p* ≥ 0.05; * *p* < 0.05; ** *p* < 0.01; *** *p* < 0.001. *N* = 4.

Next, we inhibited the NMDA receptor using the selective antagonist D-AP5 before or after stimulation of TLR8 with TL8-506 (Figure 4B). Interestingly, we observed that the inhibition of the NMDA receptor diminished the Ca^2+^ flux significantly. This result was further confirmed via MEA recordings (Figure 5A,B). The mean firing rate, mean bursting rate, and mean percentage of spikes in bursts induced by TL8-506 were reversed below baseline levels after blocking the NMDA function (Figure 5B). This suggests that TLR8 might modulate neuronal activity by potentially regulating NMDA receptor function, thereby influencing calcium influx and neuronal excitability.

### 2.3. Downstream Signaling Induced by TLR8 Stimulation in Neurons

To investigate the signal transduction pathways activated upon TLR8 activation in neurons, we examined whether it follows the canonical pathway described for immune cells. In immune cells, ligation of TLR8 triggers three major pathways: activation of the transcription factor NF-kB via degradation of the NF-κB-inhibitor IκB-α, activation of the MAPKs JNK and p38 through phosphorylation leading to activation of the transcription factor AP-1, and induction of the transcription factor IRF-7, resulting in the synthesis and release of various inflammatory species (e.g., type-I interferons, TNF-α, MIP-Iα) [24,25].

To determine the activation status of downstream proteins induced by TLR8 stimulation in neurons, we conducted immunoblot analysis using the corresponding antibodies and compared the results with those obtained from mouse macrophages. TLR4 stimulation with lipopolysaccharide (LPS) was included, as TLR4 has been reported to be activated in both immune cells and neurons [26]. TLR8 was stimulated with TL8-506 (1.0 µg/mL) for nine minutes, as this timeframe corresponded to the duration of the HD-MEA recordings. Stimulation of TLR8 in mouse macrophages led to the degradation of IkB-α, although at a lower level compared to the degradation induced by LPS stimulation (Figure 5C). In contrast, in this short timeframe, neither TLR8 nor TLR4 stimulation resulted in significant IkB-α degradation in neurons. The phosphorylation of the MAPK p38 was significantly increased after TLR8 activation, while the results were not statistically significant regarding the phosphorylation of JNK.

In conclusion, acute stimulation of TLR8 (9 min) in neurons did not lead to IkB-α degradation in an interval that allowed the initiation of this process in macrophages. In this period of time, we observed a significant increase in p38 MAPK activation.

### 2.4. Possible Mechanism of TLR8 Activation Inducing Neuronal Excitability

**Figure 6:**
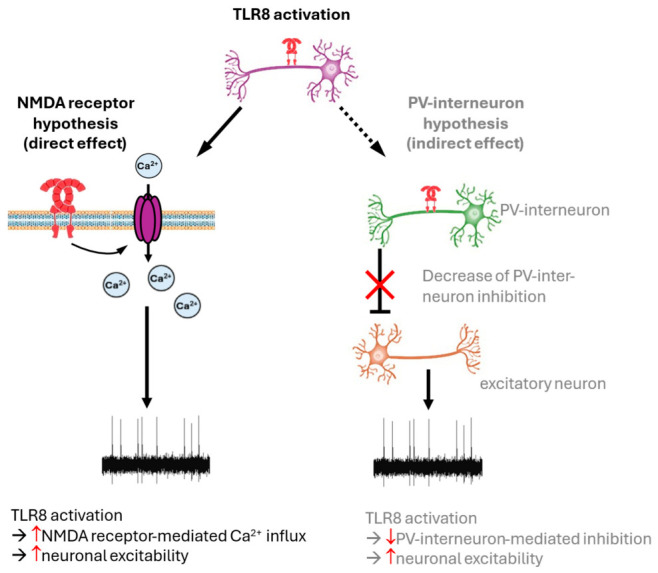
Two alternative models that might explain the observed TLR8-mediated neuronal activity. Our results suggest that TLR8 activation might induce neuronal excitability through NMDA receptor modulation (solid line). However, an alternative mechanistic hypothesis might involve the suppression of PV interneuron-mediated inhibition (broken line); this hypothesis warrants further experimental validation.

Based on the previous results, we propose a potential mechanism of action (Figure 6). It is feasible that TLR8 ligation may promote indirectly the activity of NMDA receptors leading to increased neuronal excitability. In addition, the physiological function of PV interneurons (GABAergic interneurons) is to inhibit excitatory neurons in the hippocampus [27]. It is known that focal silencing of PV interneurons in mice reduces the seizure threshold and may induce epileptogenesis [28]. While we did not explicitly record electrophysiological changes from PV neurons, a disruption to this specific population would trigger network disinhibition and an excess of background excitation, a phenomenon widely documented when PV interneuron pacing is compromised. Thus, we hypothesize a possible alternative mechanism of action whereby TLR8 activation in neuronal cultures might lead to a decrease in PV interneuron-mediated inhibition, resulting in an excess of neuronal activity.

## 3. Discussion

Previously, we reported the age-dependent expression of TLR8 in various types of neurons in mouse hippocampal brain slices [14]. In the present study, we shed some light on the functional role of neuronal TLR8 in postnatal hippocampal neuronal cultures.

The described immunocytochemistry results provide new insights into the expression of TLR8 (and TLR7) in postnatal hippocampal neurons. In an earlier study by Ma et al., TLR8 expression in embryonic cortical neurons (E16, embryonic day 16) was primarily localized to the perinuclear cytoplasm and neurites, including growth cones [9]. Postnatally, TLR8 was predominantly localized in neuronal somata. In addition, the researchers did not detect TLR7 expression in neurons. Our results point to a homogenous expression of TLR8 in hippocampal neurons (DIV14; postnatal neurons kept 14 days in vitro), which was confirmed using three distinct anti-TLR8 antibodies. Similar distribution patterns were observed in cortical neurons. Importantly, we also detected TLR7 expression in neurons and in neurospheres. However, the observed variation in the results may be attributed to differences in the ages of the animals used. We formerly reported a discrepancy to the findings of Ma et al. [9] when addressing TLR8 expression in mouse brain regions. The authors described diffuse TLR8 expression in most regions in postnatal mouse brains and mainly somatic neuronal localization in Swiss Webster mice, whereas we—using C57BL6/J mice—observed a strong expression in PV-expressing interneurons in the hippocampus (soma and processes), while principal neurons only showed faint labeling [14]. Ma et al., however, did not further investigate expression of TLR8 in distinct classes of neurons.

Traditionally, TLR3/7/8 and TLR9 were considered to be intracellular receptors expressed exclusively within the endosomal compartment [29]. However, recent studies have reported cell membrane expression, facilitated by the cysteine protease cathepsins for TLR3, TLR7, and TLR9 [30,31,32]. In our study, we have shown that TLR8 appears expressed at foci, the typical expression pattern of endosomal localization; however, we found extra-foci expression along the soma and dendritic processes. This raises the intriguing possibility that a broader expression of TLR8 in neurons might contribute to a faster signaling process compared to immune cells, where ligands first have to reach TLRs located at the endosome through endocytosis [33]. However, the specific host-derived ligands that may be recognized by TLR8 in neurons remain unknown and warrants further investigation.

In our study, we observed a fast increase (within seconds to minutes) in neuronal excitability (MEA experiments) following TLR8 ligation with the TLR8 agonist TL8-506, as indicated by the elevated mean firing rate, bursting rate, and percentage of spikes in bursts. This effect could potentially be attributed either to direct stimulation of neuronal TLR8 or to a confounding effect related to the inflammatory activity of glial cells present in the culture. Cytokine or reactive oxygen species production could be induced by glial-TLR8 stimulation via activation of NADPH oxidase as it has been shown in neutrophils [34]. However, several lines of evidence support the notion that the recorded MEA activity likely reflects TLR8 activation in neurons. Firstly, the increased electrical activity was observed within seconds of adding the TLR8-ligand, a short timeframe for the synthesis and release of cytokines, which typically might take hours until being produced and to exert their effects. Secondly, the presence of glial cells in our neuronal cell cultures at DIV14 used for the MEA experiments accounted for 53% astrocytes, 1% microglia, and 1% oligodendrocytes (Figure S5; also see [20]). This is due to the use of cytosine arabinoside (AraC), an inhibitor of cell proliferation, in the cell culture media at DIV3. Additionally, in our previous co-expression studies, we showed that TLR8 is not expressed in hippocampal astrocytes or microglia [14]. Further, the neuronal preparation used for the calcium flux experiments was done following the protocol by Brewer et al. [35] that separates, by gradient centrifugation, the different cell populations. Thirdly, we observed a fast inhibition of the stimulatory effect of TL8-506 by treating the neurons with the selective TLR8 antagonist CU-CPT9a that strengthens the case for TLR8-mediated neuronal activation. In the case of a cytokine-mediated effect, CU-CPT9a treatment would not show an inhibitory effect. Consequently, the observed excitatory effect is unlikely to be driven by glial-cell-mediated mechanisms.

We utilized the agonist TL8-506, a derivative of VTX-2337 [19], that was initially described by the supplier as a selective TLR8 ligand. However, later studies show that it can also activate mouse TLR7 [36]. Although some of the described effects might reflect partial TLR7 activation, our experiments show that selective inhibition of TLR8 decreases neuronal activity almost to basal levels.

Our results have raised the question of how the activation of a bona fide innate-immune receptor, such as TLR8, when stimulated in neurons, can increase neuronal excitability. Neuronal hyperexcitability, a common feature in various neurological disorders, often results from an imbalance between excitatory and inhibitory signals within the brain [37]. Specifically, either the excitatory signals are pathologically upregulated, or inhibitory signals are downregulated, leading to an overactive state. In the hippocampus, inhibition is primarily regulated by GABAergic interneurons [27]. Our previous work demonstrated that selective silencing of PV interneurons, a specific type of GABAergic interneurons, in the hippocampi of mice resulted in the development of epileptic seizures [28]. Interestingly, our immunostaining results revealed the co-expression of TLR8 in PV-expressing interneurons in postnatal hippocampal cultures at DIV14. Thus, we hypothesize that TLR8 stimulation might interfere with the GABAergic inhibition mediated by PV-specific interneurons, thereby causing, through disinhibition, an excessive increase in neuronal activity in TLR8 stimulated hippocampal cultures. However, this hypothesis needs additional experimental confirmation. Furthermore, previous studies showed that TLR4 activation in neurons leads to the phosphorylation of NMDA receptors (NR2B), resulting in an increased influx of calcium ions [11]. Thus, we question whether a similar direct activation of NMDA receptors might occur in the case of TLR8 stimulation.

Our investigation into the signaling cascade activated by neuronal TLR8 ligation revealed slight differences from the canonical pathway activated in immune cells. We observed that TLR8 activation in neurons led to the phosphorylation of the MAPK p38, which is consistent with the response observed in immune cells. However, we did not observe a significant degradation of IkB-α upon TLR8 or TLR4 stimulation in neurons in the interval tested. One possible explanation is that the degradation of IkB-α in neurons occurs at a later time point as has been described for other cell types (e.g., THP-1) [38], and the nine minutes stimulation period was not enough to reach this effect. Alternatively, the activation of TLR8 on the cell surface of neurons, as opposed to the endosomal compartment in immune cells, may trigger different intracellular signaling events leading to distinct activation effects.

In summary, we have provided evidence for the expression of TLR8 and TLR7 in postnatal hippocampal neurons. Based on our findings, we propose that neuronal TLR8 ligation increases excitability either by enhancing NMDA receptor function or by suppressing inhibitory PV-expressing interneurons. However, the multi-electrode array cultures are mixed and a fast glial ROS effect on neighboring neurons cannot be formally excluded. Thus, further experimental validation is necessary to confirm the proposed hypotheses. Our results contribute to a deeper understanding of the potential role of TLR8 in modulating neuronal activity and the intricate crosstalk between the immune and nervous systems.

## 4. Materials and Methods

### 4.1. Mice

C57BL/6J wild-type male and female mice were utilized for all the experiments. The mice were housed in single-ventilated cages and had access to food and water ad libitum.

### 4.2. Preparation of Postnatal Hippocampal Neuronal Cultures and Neurospheres

Neuronal hippocampal cultures were prepared from hippocampi obtained from mice pups aged 0–3 days (postnatal day P0–P3), following a slightly modified protocol earlier described [20]. Briefly, mouse pups were decapitated, and their brains were carefully removed to isolate the hippocampi. The collected hippocampi were dissected in ice-cold Hibernate medium (ThermoFischer Scientific, Waltham, MA, USA) supplemented with Penicillin and Streptomycin (ThermoFischer Scientific).

To dissociate the cells, the hippocampal tissue was then incubated in 1 mg/mL Papain solution (P4762; Sigma-Aldrich, St. Louis, MO, USA) at 37 °C for 10 min. Subsequently, the tissue was further incubated with 0.1 mg/mL of DNAase (Roche, Basel, Switzerland) for 5 min at 37 °C to eliminate extracellular DNA. The papain reaction was halted using CM+ medium, which consisted of Neurobasal-A (ThermoFisher Scientific) supplemented with 2% B27, 0.25% Glutamax, 0.25% L-Glutamine, 10% FBS, and 1% Penicillin/Streptomycin [20].

To obtain a cell suspension, mechanical dissociation was performed using serological pipettes, and the cells were purified by centrifugation through a 4% BSA cushion (Sigma-Aldrich) at 70× *g* for 5 min at room temperature. The purified hippocampal cells were then seeded onto MEA BioChips (coated with 0.1 mg/mL Poly-D-Lysine; Gibco, Waltham, MA, USA) at a density of 75,000 cells/chip for electrophysiological recordings (see electrophysiology), or onto pre-coated coverslips at a density of 100,000 cells/well for immunocytochemistry (24-well plates) or 160,000 cells/well for Western blot.

For culture maintenance, the cells were incubated at 37 °C and 5% CO_2_. From the time of seeding until DIV3 (days in vitro), the cells were cultivated in CM+ medium. To limit glial proliferation, at DIV3, 15% of the medium was replaced with AraC (0.3 µM, Sigma-Aldrich) and Amphotericin B (0.3 µg/mL Sigma-Aldrich) dissolved in CM- medium (BrainPhys^TM^ Neuronal medium; Stemcell Technologies, Vancouver, BC, Canada). At DIV7, 30% of the medium was replaced with CM- medium and neuronal cultures at DIV14 were considered mature for the experiments.

To isolate postnatal neurosphere-derived neurons hippocampal tissue was dissociated with Trypsin, and neurons were separated by forming a gradient with OptiPrep (Sigma-Aldrich) [35]. From the obtained cell layers, the neuronal cell suspension was collected and cultured in T75 flasks in Neurobasal-A media, with B27 supplement and growth factors FGF-2 (20 ng/mL) and PDGF (20 ng/mL) (Invitrogen, Carlsbad, CA, USA) for two weeks. The media was renewed every 3 to 4 days. Afterwards, the cells were seeded in poly-lysine-coated glass coverslips in 24-well plates in Neurobasal-A media and B27 without growth factors. The neurospheres differentiated into adherent neurons for two weeks and immunocytochemistry with anti-TLR7 and anti-TLR8 antibodies was performed as indicated. For the calcium flux experiments, the layer of neurons separated in the gradient indicated above was plated in poly-lysine coated plates and they were differentiated during two weeks in Neurobasal media with B27 supplement without growth factors. The media was renewed every 3 to 4 days.

### 4.3. Immunocytochemistry

Immunocytochemistry was performed on postnatal hippocampal neurons at DIV14, using the cell preparation methods described above. The cells were washed with phosphate-buffered saline (PBS) and fixed with 4% paraformaldehyde (PFA; Carl Roth, Karlsruhe, Germany) for 10 min. After fixation, the cells were washed three times for 5 min with PBS and permeabilized with 0.1% Tween20 (Roth) in PBS for 10 min. Subsequently, cells were washed with wash buffer (PBS with 0.05% Tween) and incubated with blocking serum (1% normal goat serum, NGS, Sigma-Aldrich) in wash buffer for 1 h. The previous steps were done at room temperature.

Primary antibodies (Table S1) were diluted in diluent assay (1% NGS, 0.05%, Tween20, 0.1% sodium azide), and the cells were incubated with the primary antibodies overnight at 4 °C. Afterwards, the cells were washed three times with wash buffer for 5 min each. Then, the secondary antibody (Table S1) diluted in diluent assay was added and incubated for 2 h at room temperature. The cells were rinsed with wash buffer, and for nuclear staining, the cells were incubated for 1 min with Hoechst dye (1:40,000, 10 mg/mL; CST 4082S) and washed with PBS. Coverslips were mounted with Mowiol (RT-SZ2X-03; Abcam, Cambridge, UK), and pictures were taken using an Olympus IX73 microscope at a magnification of 20× or 60× with an oil immersion objective. The images were analyzed with ImageJ/Fiji software (FijiWin64/ImageJ2015; National Institutes of Health, USA). The controls of antibody-specificity used in the immunocytochemistry are shown in Figure S1B,C.

### 4.4. Multi-Electrode Array (MEA)

MEA BioChip preparation

For electrophysiological recordings, postnatal hippocampal cultures were cultivated on high-density multi-electrode array (HD-MEA) Accura Chips (3Brain AG, Zürich, Switzerland). These chips consist of 4096 recording electrodes arranged in a 64 × 64 grid, with each electrode measuring 21 × 21 µm^2^ and a pitch of 60 µm. The active area of the chip is 3.84 mm × 3.84 mm. Prior to cell plating, the electrode region of the MEA chips was sterilized with 70% ethanol for 30 min, while the outer surface was cleaned with 96% ethanol and then air-dried under a sterile hood.

Sterilized MEA BioChips were coated with 0.1 mg/mL Poly-D-Lysine (Gibco) and incubated for 3 h at 37 °C with 5% CO_2_. Afterwards, the chips were washed with sterile water, and they were covered with a petri dish. Cells from the hippocampal dissociation process were seeded at a concentration of 75,000 cells per MEA Chip in a 150 µL drop onto the electrode area of the chips. After the cells settled (2–3 h), 1.5 mL CM+ medium was added, and they were incubated at 37 °C with 5% CO_2_. The electrophysiological activity was recorded at DIV13–14 when the connectivity between the neurons is at its highest level [20].

MEA recordings

Extracellular recordings were conducted using the BioCAM Duplex device and the data were collected with the Brainwave4 software (3Brain AG, Zürich, Switzerland). The MEA chips were inserted in the pre-heated (37 °C) BioCAM Duplex device, and the volume of the culture medium in the chip was adjusted to 1 mL. Firstly, the baseline activity of the neuronal cultures was recorded for 2–3 min. Then, each compound (see Table 1) was added to the medium, and neuronal activity was recorded for 3 min for each drug concentration and compared to the unstimulated baseline activity. At the end of each recording, the voltage-gated sodium channel blocker tetrodotoxin (TTX citrate, 1 µM, HelloBio, Bristol, UK) was added to the MEA chip. Electrodes that still fired after TTX application (>0.5 spikes/s) were considered defective and excluded from further analysis.

MEA Data Analysis

Data analysis was performed using the Brainwave4 software (3Brain AG, Zürich, Switzerland). Spike detection was performed using the Precise Timing Spike Detection (PTSD) algorithm, with the spike detection threshold set at 9 times the standard deviation of the raw signal. Bursts were defined as having five or more spikes within a 30 ms interval. Only electrodes with a firing rate above 0.05 spike/s during the baseline activity were considered active electrodes and included in the analysis. Mean firing rate (spikes/s), mean bursting rate (bursts/min), and mean percentage of spikes in bursts (%) were calculated for each MEA recording using Brainwave4 (3Brain AG). The data was normalized to the mean baseline activity of each MEA recording to account for any inherent variability among different recordings.

### 4.5. Calcium Imaging of Primary Hippocampal Neurons

Primary hippocampal neurons were isolated from postnatal day 1 (P1) pups [35], and maintained in vitro for 14 days at 37 °C in a humidity-controlled atmosphere containing 5% CO_2_. Three hours prior to imaging, the culture medium in 96-well plates was adjusted to a final volume of 100 µL per well. Calcium imaging was performed using the Fluo-4 NW Calcium Assay Kit (Thermo Fisher Scientific) according to the manufacturer’s protocol. For this assay, Fluo-4 NW dye-loading solution containing 2.5 mM probenecid was prepared. The neurons were pre-incubated for 30 min at 37 °C with the inhibitors D-AP5 (5 µg/mL) or CU-CPT9a (0.1 µM). To control potential vehicle-related effects of CU-CPT9a, an equivalent volume of the corresponding vehicle solution was applied to the control wells. Following the inhibition period, the cells were stimulated with TL8-506 (500 ng/mL) or vehicle. Immediately after addition of the stimulant, 1× dye-loading solution was added, and the cells were incubated for 5 min at 37 °C to allow intracellular de-esterification of the calcium indicator. At the end of the experiment, ionomycin (2 µM) was added to record total fluorescence. Fluorescence images were acquired immediately after the incubation periods using an epifluorescence microscope controlled by Leica Application Suite X software (LAS X, 1.5.1.13187; Leica Microsystems, Wetzlar, Germany) and equipped with a Leica DFC3000 G camera (Leica Microsystems), a GFP filter set, and a 10× objective. All images were obtained using identical exposure times and acquisition parameters across conditions, and image acquisition was completed within minutes of dye loading to capture stimulus-associated calcium signals. Experiments were performed on three independent culture plates, with three technical replicate per condition. Quantitative analysis of the fluorescence signals was conducted using ImageJ Fiji. Regions of interest (ROIs) corresponding to individual neuronal somata were identified on the fluorescence images and the mean fluorescence intensity extracted for each ROI. The resulting intensity values were then used to compare calcium-dependent fluorescence between the stimulated, inhibited, vehicle-treated, and unstimulated groups.

### 4.6. Western Blot

Postnatal hippocampal cultures were cultured on pre-coated coverslips until DIV14 as described above. After stimulating the neurons with TL8-506 (1 µg/mL) or LPS (5 µg/mL), the cells were washed with PBS and lysed with lysis buffer (PBS, 2.5% TritonX, protease inhibitors 1:10 (Sigma), phosphatase inhibitors 1:10 (Sigma). As a control, immortalized bone-marrow-derived macrophages (kindly provided by D.T. Golenbock, Worcester, MA, USA) [39] were grown overnight in a 24-well plate at a density of 150,000 cells/well. The next day, they were stimulated (nine minutes) and lysed in the same manner as the neuronal cultures. The lysates from both neurons and macrophages were cleared by centrifugation at 1110× *g* for 10 min at 4 °C, and equal amounts of the obtained supernatants were loaded and separated by electrophoresis on SDS-PAGE acrylamide gels (10%, 120 V, for 1 h). After separation, the proteins were transferred onto a PDVF membrane (0.45 µm, Carl-Roth) in a semi-dry device (12 V, 75 min; PeqLab, Erlangen, Germany). The membrane was then blocked with 2.5% BSA (dissolved in TBS-T: 20 mM Tris-buffer pH 7.4, 0.15 M NaCl, 0.1% Tween20) for 1 h and subsequently incubated overnight at 4 °C with the primary antibody solution (Table S1) diluted in blocking solution. Then, the membrane was washed with TBS-T and it was incubated with the secondary antibody for 2 h at room temperature (Table S1). Detection was performed using HRP-substrate and the imaging was done using the Fusion program (Fusion FX7, PeqLab). Image analysis was done with ImageJ/Fiji (FijiWin64/ImageJ2015) and FusionCapt software version SL416.02 (PeqLab). Normalization was performed by dividing the mean gray intensity of each band through the mean gray intensity of the loading control β-Actin. Then, the values were normalized to the non- stimulated conditions.

### 4.7. Statistical Analysis

The results are presented as mean values ± standard error of the mean (SEM). Statistical analysis was conducted using GraphPad Prism9 software (GraphPad, San Diego, CA, USA). One-way analysis of variance (ANOVA) was used to compare the data, followed by Dunnett’s post hoc test for multiple comparisons. *p*-values equal to or less than 0.05 are denoted with *, *p*-values equal to or less than 0.01 are denoted with **, *p*-values equal to or less than 0.001 are denoted with ***, and *p*-values equal to or less than 0.0001 are denoted with ****. For immunoblot analysis to compare two samples, Student’s *t*-test was used.

## Figures and Tables

**Table 1:** Compounds used during the multi-electrode array experiments and where indicated.

Compound	Mode of Action	Manufacturer	Catalogue Number	Solvent
TL8-506	TLR8 agonist	Invivogen	tlrl-tl8506	H_2_O
CU-CPT9a	TLR8 antagonist	Invivogen	inh-cc9a	CU-CPT9a solvent
Tetrodotoxin (TTX)	Voltage-gated sodium channel blocker	HelloBio	HB1035	H_2_O
D-AP5	NMDA-receptor antagonist	HelloBio	HB0225	H_2_O

## Data Availability

The original contributions presented in this study are included in the article/Supplementary Materials. Further inquiries can be directed to the corresponding author.

## Supplementary Materials

The following supporting information can be downloaded at: https://www.mdpi.com/article/10.3390/ijms27146232/s1.

## Abbreviations

AMPAR1: a-amino-3-hydroxy-5-methyl-4-isoxazolepropionic acid receptor 1. AraC: cytosine arabinoside. BS: Baseline; DAMPs: danger-associated molecular patterns; DIV: days in vitro. E: embryonic. EEA1: Early endosome antigen 1. HD-MEA: high-density multi-electrode array. Map2: microtubule-associated protein 2. NMDA: N-methyl-D-aspartate. P: postnatal. PAMPs: pathogen-associated molecular patterns. PRRs: pattern-recognition receptors. PV: parvalbumin. TLRs: Toll-like receptors. TTX: tetrodotoxin.
