# Supplementary material for: Toll-like Receptor 8 (TLR8) Is Expressed in Mouse Hippocampal Neurons and Modulates Neuronal Excitability
Source: Int J Mol Sci. 2026 Jul 13;27(14):6232. doi: 10.3390/ijms27146232 (PMC13411007; doi:10.3390/ijms27146232)
Supplement: Supplementary file 1 [file ijms-27-06232-s001.zip › ijms-4262426-supplementary.pdf]

International Journal of
Molecular Sciences

Figure S1A. TLR8 staining (red) with different antibodies (Sigma-Merck) and the secondary antibody anti-rabbit 647 (4414S Cell Signaling) and Hoechst (4082S Cell Signaling).

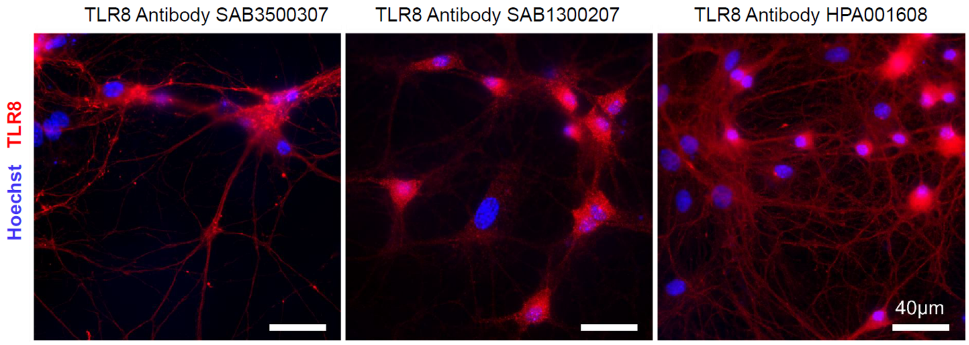

Figure S1B. Validation of TLR7 and TLR8 antibody specificity.-Immunostaining of wild-type and TLR8 knock-out fibroblasts using anti-TLR8 antibody (Sigma, SAB1301375). A strong TLR8 signal was detected in wild-type cells, while TLR8 knockout cells showed only weak background staining.-Immunostaining of wild-type and TLR7 knockout fibroblasts using an anti-TLR7 antibody (Sigma, WH0051284M4) shows a loss of TLR7 signal in the knock-out cells.-The specificity of an additional TLR8 antibody (Sigma, SAB3500307) used to label neurons in Supplementary 1A, was tested in wild-type and TLR8 knock-out fibroblast.-Negative control: wild-type fibroblasts stained with secondary antibody. For all experiments, 1×10^5 cells were seeded per condition and transfected with 2 µg of MISSIONesiRNA mouse TLR7 or MISSIONesiRNA mouse TLR8 (Sigma Merck) and Fugene (Promega) for two days.

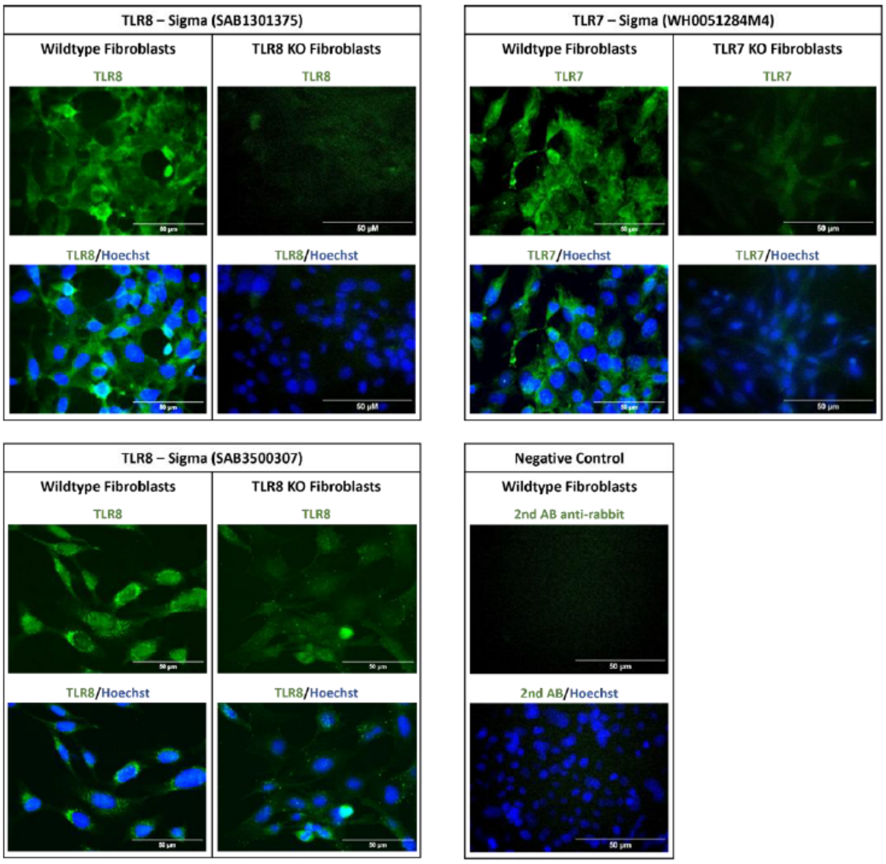

Figure S1C. Primary hippocampal neurons from P1 pups were cultured for 2 weeks at 37 °C and stained with secondary antibodies only. No specific signal was detected.

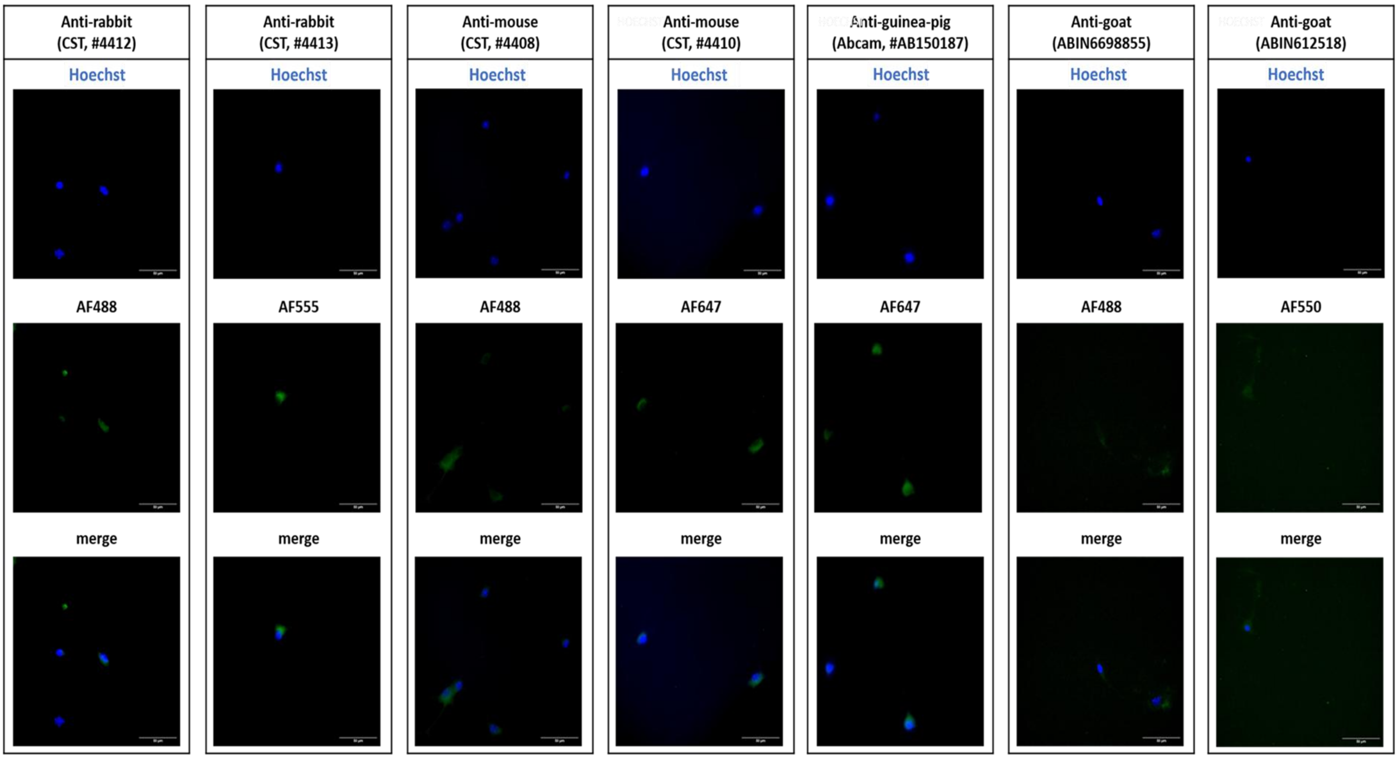

Figure S2. (**A**) Immunohistochemistry of TLR8 (green) in cultured postnatal hippocampal neurons at DIV14, along with Map2 (red) as a neuronspecific marker. The cell nuclei were stained with HOECHST (blue). (**B**) Immunohistochemistry of TLR7 (gray) in cultured postnatal hippocampal neurons at DIV14 along with Map2 (red). Cell nuclei were stained with HOECHST (blue). The scale bar represents 50 µm.

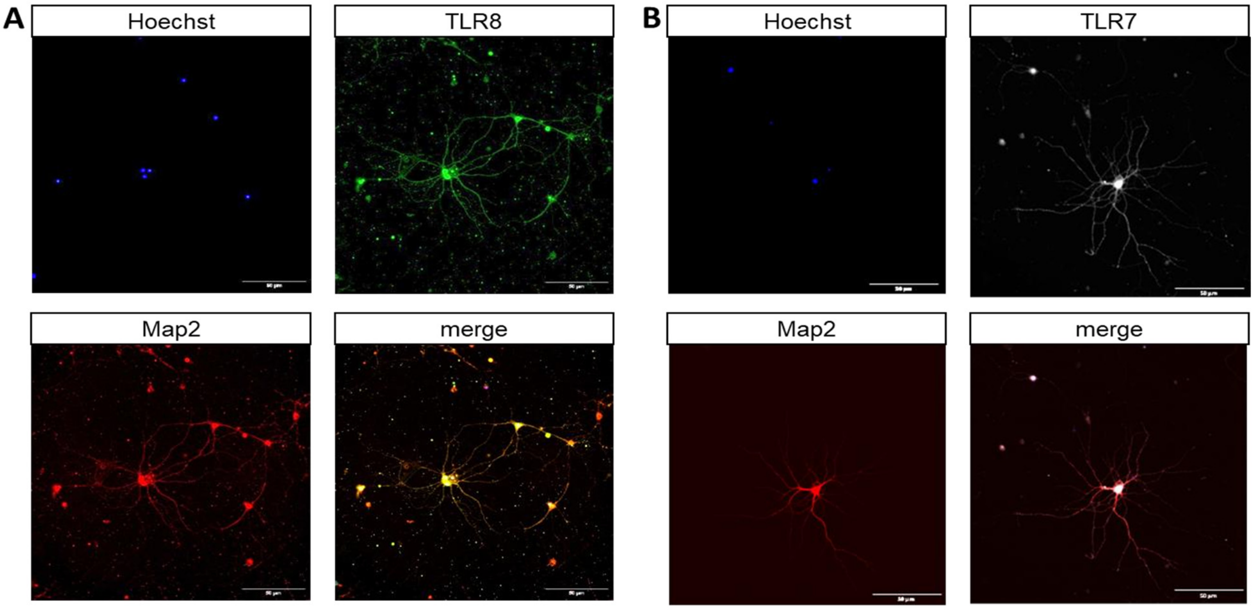

Figure S3. Neurons derived from neurospheres after two weeks of culture in differentiation media. TLR8 and TLR7 (green) are expressed in soma and dendrites. Nuclei (blue).

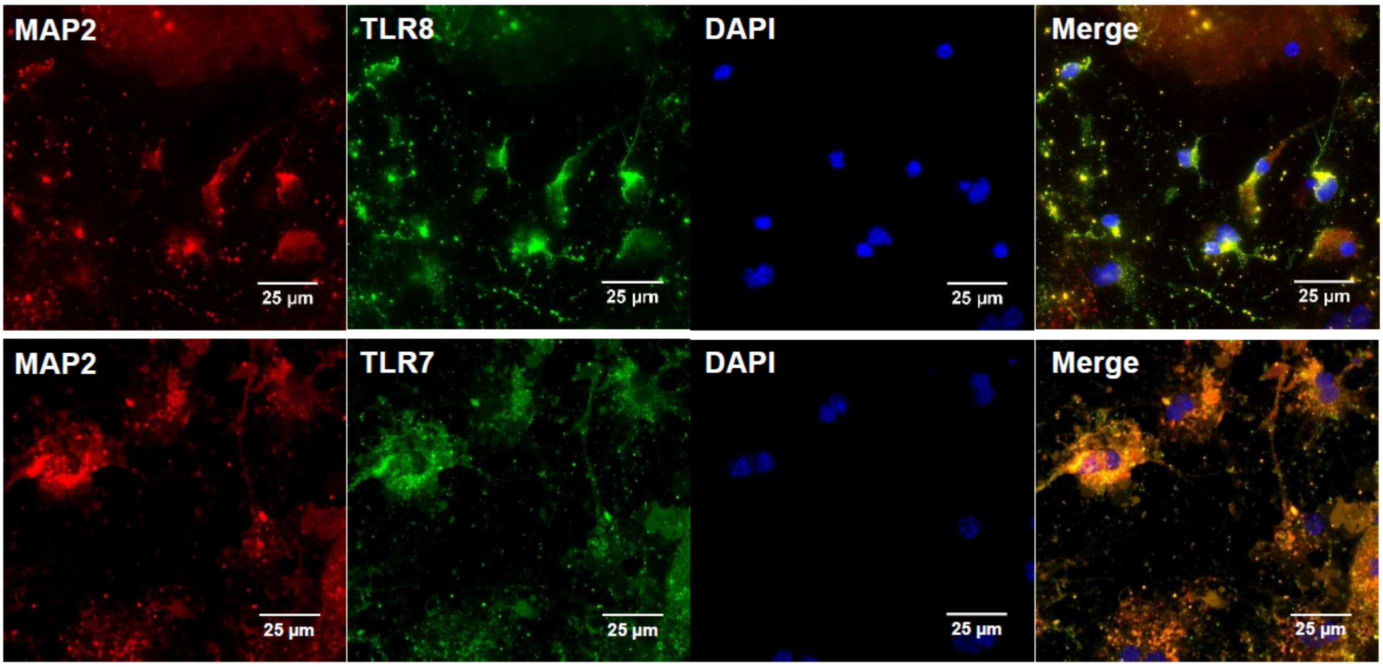

Figure S4. ImmunocytochemistryexperimentsshowingTLR8expressioninprimarymousehippocampalneuronsandinPVneurons.

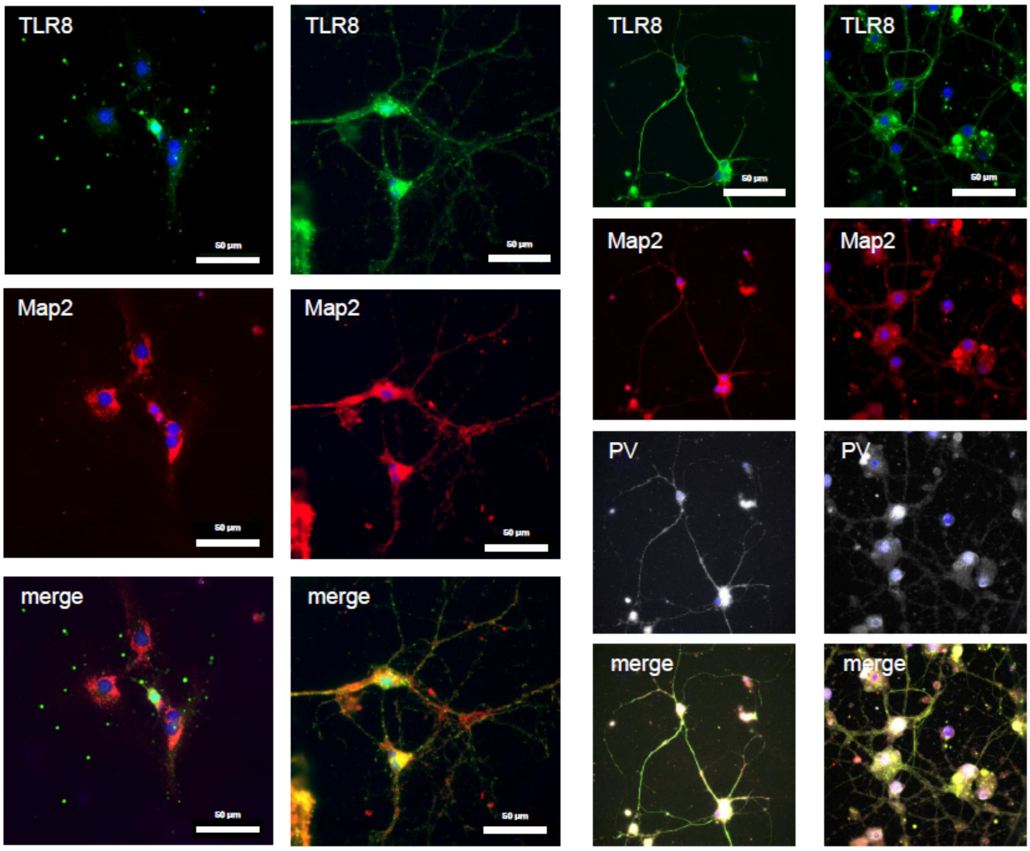

Figure S5. Cellular composition of postnatal hippocampal cultures. Representative immunocytochemistry images from **(A)** DIV7, DIV14 and DIV21 cultures stained with antibodies against GFAP (astrocytes, red), MAP2 (neurons, green), **(B)** DIV14 culture stained with antibodies against CD11b (microglia, yellow) and MOG (oligodendrocytes, red). Nuclei in (**A**) and (**B**) are stained with Hoechst. (**C**) Overall cellular composition of the cultures including astrocytes, neurons, microglia, and oligodendrocytes at DIV14. (**D**) TLR8 (green) expression in astrocytes using the marker GFAP (red). n = 5 random fields from N = 3 independent cultures. Scale bar in (**A**) = 100 µm. Scale bar in (**B**), top row = 100 µm; bottom row = 50 µm.

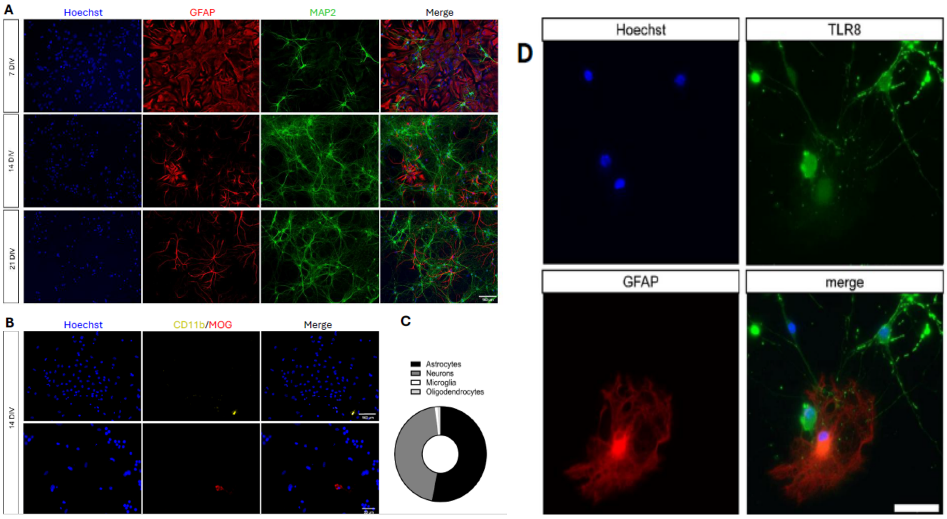

https://doi.org/10.3390/ijms27146232

Figure S6. Validation of electrophysiological responses of hippocampal postnatal neuronal cultures grown on MEA chips. Electrical activity of the cultures was increased by a 3-minute incubation with the GABAA receptor antagonist picrotoxin (PTX,100μM), followed by a 3-minute incubation with the antiseizure medications (A) topiramate (100μM), (B) tiagabine(50μM),and(C)levetiracetam (150μM). Incubation with picrotoxin increased mean firing rate, mean bursting rate, and mean percentage of spikes in bursts. The increased electrophysiological activity of the cultures was reversed by thefast-acting antiseizure drugs topiramate and tiagabine, while the slow-actingdrug levetiracetam failed to reverse the activity in the 3minutes incubation period.Results are represented as mean±SEM, n=3 independent recordings from N = 3independent cultures.One-wayANOVA corrected for multiple comparisons by Tukey's test indicatessignificance (ns=notsignificant,*p<0.05,**p<0.01,***p<0.001,****p<0.0001).

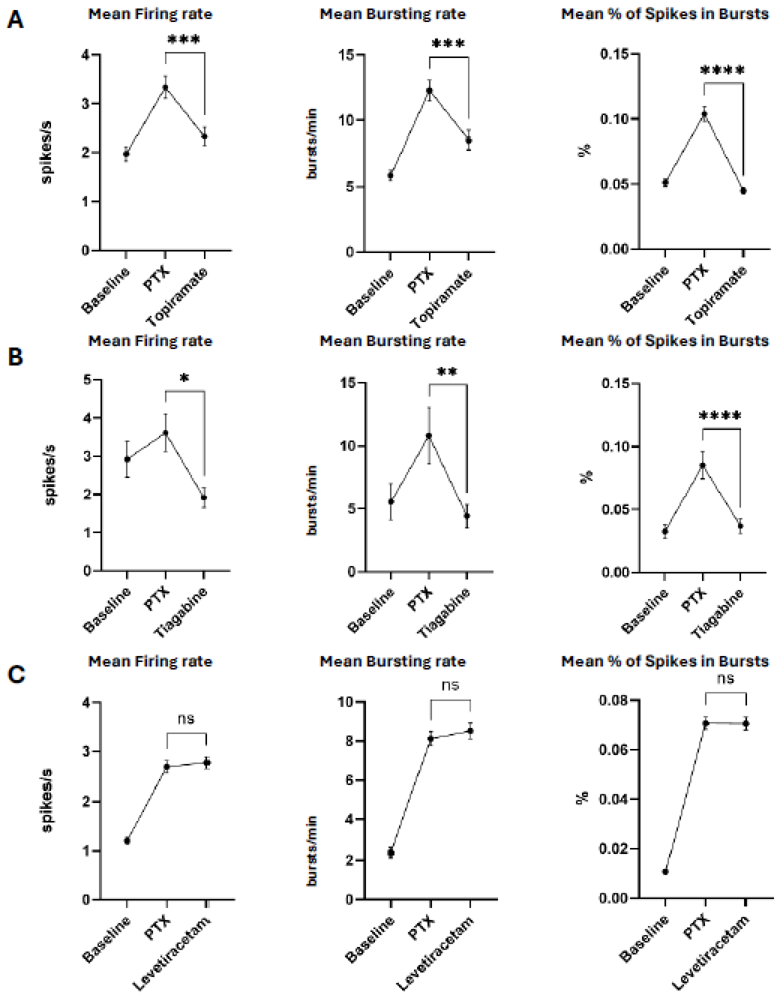

Table S1. Antibodies used for immunocytochemistry and for Western-blot.

**Primary antibodies IHC**	**Species**	**Manufacturer**	**Catalogue number**
EEA1	Guinea pig	Synaptic Systems	237105
Map2	Goat	Novusbio	NBP3-05552
Map2	Rabbit	Cell Signaling Technology	8707
TLR7	Mouse	Sigma	WH0051284M4
TLR8	Mouse	Sigma	SAB1301375
TLR8	Rabbit	Sigma	SAB3500307
TLR8	Rabbit	Sigma	SAB1300207
TLR8	Rabbit	Sigma	HPA001608
PV	Guinea pig	Synaptic Systems	195004
PV	Mouse	Sigma-Aldrich	P3088
CD11B	Rat	Cell Signaling Technology	465125
GFAP	Mouse	Sigma	3670S
GFAP-Cy3	Mouse	Sigma	C9205
Iba1	Rabbit	Cell Signaling Technology	17198S
MOG	Mouse	Sigma-SAB	MAB5680
**Secondary antibodies IHC**	**Species**	**Manufacturer**	**Catalogue number**
Anti-goat IgG (DyLightTN 488)	Rabbit	Antikoerper-online	ABIN6698555
Anti-goat IgG (DyLightTN 550)	Rabbit	Antikoerper-online	ABIN612518
Anti-guinea pig IgG H&L (Alexa Fluor® 647)	Goat	Abcam	AB150187
Anti-mouse IgG (H+L), F(ab') Fragment (Alexa Fluor® 488)	Goat	Cell Signaling Technology	4408
Anti-mouse IgG (H+L), F(ab') Fragment (Alexa Fluor® 647)	Goat	Cell Signaling Technology	4410
Anti-rabbit IgG (H+L), F(ab') Fragment (Alexa Fluor® 555)	Goat	Cell Signaling Technology	4413
Anti-rabbit IgG (H+L), F(ab') Fragment (Alexa Fluor® 488)	Goat	Cell Signaling Technology	4412
**Primary antibodies WB**	**Species**	**Manufacturer**	**Catalogue number**
IκB-α	Mouse	Cell Signaling Technology	4814
p-IRF-7	Rabbit	Cell Signaling Technology	24129S
p-p38	Rabbit	Cell Signaling Technology	4511T
p-JNK	Rabbit	Cell Signaling Technology	4668T
β-Actin	Mouse	Cell Signaling Technology	3700S
**Secondary antibodies WB**	**Species**	**Manufacturer**	**Catalogue number**
Anti-mouse HRP	Horse	Cell Signaling Technology	7076P2
Anti-rabbit HRP	Goat	Cell Signaling Technology	7074P2

https://doi.org/10.3390/ijms27146232
